# Invasive non‐native species likely to threaten biodiversity and ecosystems in the Antarctic Peninsula region

**DOI:** 10.1111/gcb.14938

**Published:** 2020-01-13

**Authors:** Kevin A. Hughes, Oliver L. Pescott, Jodey Peyton, Tim Adriaens, Elizabeth J. Cottier‐Cook, Gillian Key, Wolfgang Rabitsch, Elena Tricarico, David K. A. Barnes, Naomi Baxter, Mark Belchier, Denise Blake, Peter Convey, Wayne Dawson, Danielle Frohlich, Lauren M. Gardiner, Pablo González‐Moreno, Ross James, Christopher Malumphy, Stephanie Martin, Angeliki F. Martinou, Dan Minchin, Andrea Monaco, Niall Moore, Simon A. Morley, Katherine Ross, Jonathan Shanklin, Katharine Turvey, David Vaughan, Alexander G. C. Vaux, Victoria Werenkraut, Ian J. Winfield, Helen E. Roy

**Affiliations:** ^1^ British Antarctic Survey Natural Environment Research Council Cambridge UK; ^2^ UK Centre for Ecology & Hydrology Oxfordshire UK; ^3^ Research Institute for Nature and Forest (INBO) Brussels Belgium; ^4^ Scottish Association for Marine Science Scottish Marine Institute Dunbeg UK; ^5^ GB Non‐native Species Secretariat Animal and Plant Health Agency York UK; ^6^ Environment Agency Austria Vienna Austria; ^7^ Department of Biology University of Florence Florence Italy; ^8^ Falkland Islands Government Stanley Falkland Islands; ^9^ Government of South Georgia & the South Sandwich Islands Stanley Falkland Islands; ^10^ Department of Biosciences Durham University Durham UK; ^11^ SWCA Environmental Consultants Honolulu HI USA; ^12^ Sainsbury Laboratory University of Cambridge Herbarium Cambridge University Cambridge UK; ^13^ CABI Egham UK; ^14^ Department of Forest Engineering ERSAF University of Córdoba Córdoba Spain; ^15^ National Agri‐Food Innovation Campus Fera Science Ltd. York UK; ^16^ The Administrator's Office Government of Tristan da Cunha Edinburgh of the Seven Seas Tristan da Cunha; ^17^ The Cyprus Institute Nicosia Cyprus; ^18^ Marine Organism Investigations Killaloe Ireland; ^19^ Directorate Environment and Natural Systems of the Lazio Regional Authority Rome Italy; ^20^ Falklands Conservation Stanley Falkland Islands; ^21^ Medical Entomology Group Emergency Response Science & Technology Public Health England Salisbury UK; ^22^ Laboratorio Ecotono Centro Regional Universitario Bariloche Universidad Nacional del Comahue/INIBIOMA‐CONICET Bariloche Argentina

**Keywords:** biodiversity, horizon scanning, non-native, pathways, Protocol on Environmental Protection to the Antarctic Treaty, risk assessment

## Abstract

The Antarctic is considered to be a pristine environment relative to other regions of the Earth, but it is increasingly vulnerable to invasions by marine, freshwater and terrestrial non‐native species. The Antarctic Peninsula region (APR), which encompasses the Antarctic Peninsula, South Shetland Islands and South Orkney Islands, is by far the most invaded part of the Antarctica continent. The risk of introduction of invasive non‐native species to the APR is likely to increase with predicted increases in the intensity, diversity and distribution of human activities. Parties that are signatories to the Antarctic Treaty have called for regional assessments of non‐native species risk. In response, taxonomic and Antarctic experts undertook a horizon scanning exercise using expert opinion and consensus approaches to identify the species that are likely to present the highest risk to biodiversity and ecosystems within the APR over the next 10 years. One hundred and three species, currently absent in the APR, were identified as relevant for review, with 13 species identified as presenting a high risk of invading the APR. Marine invertebrates dominated the list of highest risk species, with flowering plants and terrestrial invertebrates also represented; however, vertebrate species were thought unlikely to establish in the APR within the 10 year timeframe. We recommend (a) the further development and application of biosecurity measures by all stakeholders active in the APR, including surveillance for species such as those identified during this horizon scanning exercise, and (b) use of this methodology across the other regions of Antarctica. Without the application of appropriate biosecurity measures, rates of introductions and invasions within the APR are likely to increase, resulting in negative consequences for the biodiversity of the whole continent, as introduced species establish and spread further due to climate change and increasing human activity.

## Graphical abstract

The Antarctic Peninsula region (APR) is increasingly vulnerable to biological introductions and invasions, caused by increases in the intensity, diversity and distribution of human activities. This study identified 103 species that are likely to present a risk to biodiversity and ecosystems within the APR over the next 10 years; marine invertebrates dominated the list of highest risk species, but vertebrate species were thought unlikely to establish within the 10 year timeframe. We recommend the further development and application of biosecurity measures in the APR, and further studies to identify potential biological invaders in other regions of Antarctica.

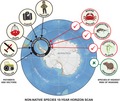

See also the Commentary by https://doi.org/10.1111/gcb.15013

## 1 INTRODUCTION

**Figure 1:**
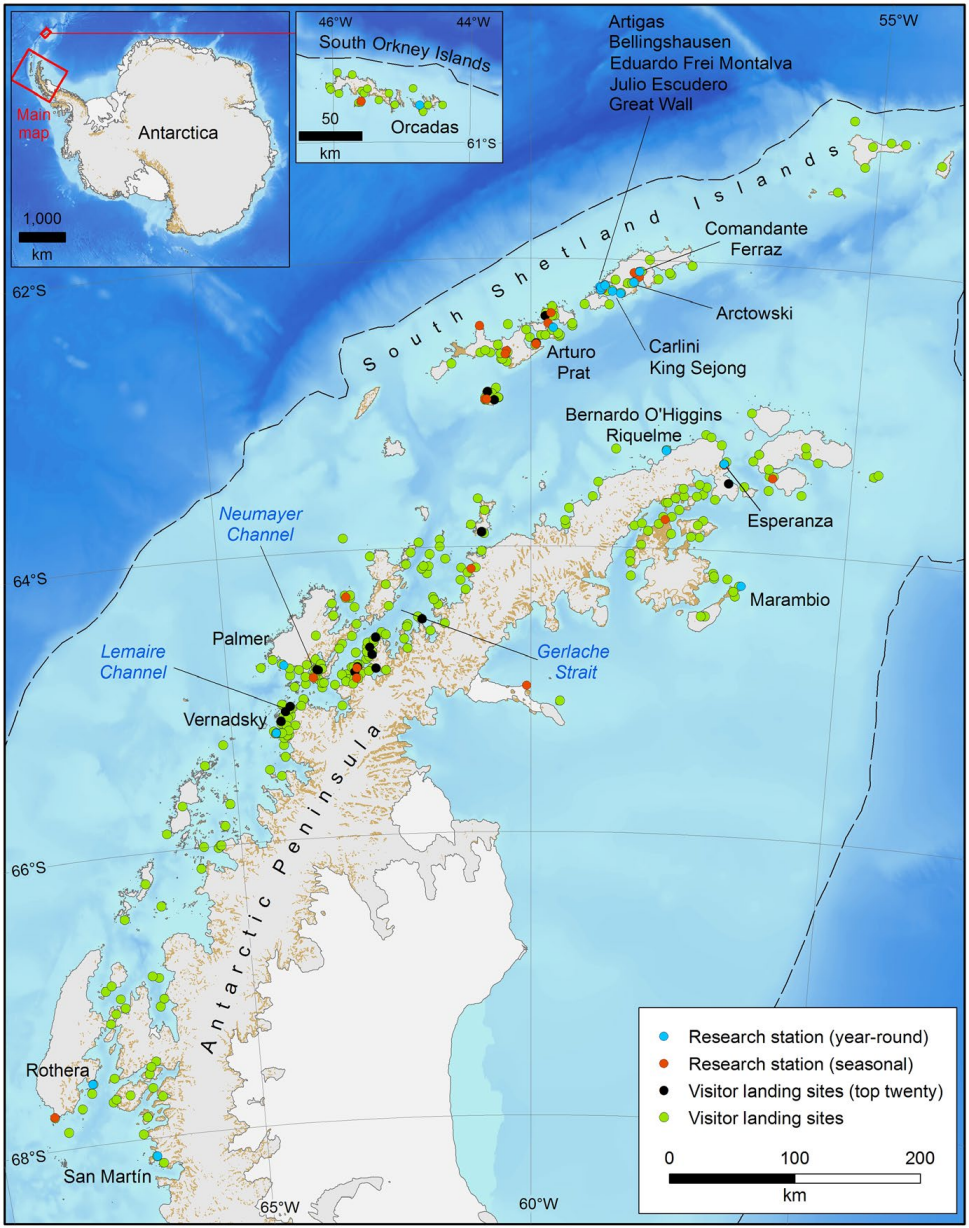
Map of the Antarctica Peninsula region showing the location of major research stations and infrastructure and tourist visitor sites. The dashed line indicates the edge of the continental shelf at 1,000 m depth

Few areas of the world are as remote and little impacted by humans as Antarctica, yet the biodiversity of the continent is under increasing threat from invasive non‐native species (Frenot et al., 2005; Hughes & Convey, 2010; Tin, Liggett, Maher, & Lamers, 2014). Here we consider the species to be invasive when their presence causes negative impacts upon the ecosystem to which they are introduced. Within the Antarctic Treaty area (the area south of 60°S), the Antarctic Peninsula region (APR), which encompasses the Antarctic Peninsula, South Shetland Islands and South Orkney Islands (Figure 1), is predicted to be at greatest risk (Chown et al., 2012). This is because it is: (a) the closest to another continent (South America); (b) the least climatically extreme region, with the highest summer temperatures and the longest growing season; (c) the largest focus of human activity (both governmental and tourism); and (d) the region which has experienced the largest rise in temperatures since the 1950s, this being predicted to continue (Bellard et al., 2013; Bracegirdle, Connolley, & Turner, 2008; Pertierra, Hughes, Vega, & Olalla‐Tárraga, 2017; Siegert et al., 2019; Turner et al., 2016). Currently all 14 of the known non‐native species within the Antarctic Treaty area are found within the APR, which demonstrates the vulnerability of the region to introductions (Chown et al., 2012; Hughes, Pertierra, Molina‐Montenegro, & Convey, 2015; Huiskes et al., 2014).

The mountainous Antarctic Peninsula has an area of c. 420,000 km^2^ and stretches for 1,300 km from c. 74 to 63°S towards South America, 1,000 km distant across the Drake Passage. The mostly submarine Scotia Arc, linking Antarctica and southern South America, includes the South Shetland Islands, South Orkney Islands and, further north, the South Sandwich Islands and the sub‐Antarctic island of South Georgia. In contrast with the colder Antarctic continent, the APR has a milder maritime climate, with coastal areas experiencing mean air temperatures around 0–3°C during the summer months (Walton, 1984). Snow and permanent ice covers c. 97% of the area, and terrestrial communities are restricted to areas of ice‐free ground, separated by permanent ice or ocean on a scale of metres to tens of kilometres. The indigenous flora of the APR includes two higher plants, various bryophytes (c. 125 species) and eukaryotic algae (Convey, 2017). The area also contains c. 250 lichen species (Convey, 2017; Øvstedal & Smith, 2001). Terrestrial vertebrates are absent (other than sheathbills, *Chionis albus*, as scavengers associated with seal and penguin colonies), and faunal diversity consists of Diptera (two species), microarthropods (46), nematodes (>200), tardigrades (26), rotifers (>50) and protozoa (Convey, 2017). Diversity decreases with increasing latitude and altitude, with microbial communities becoming increasingly dominant as environmental conditions become more extreme. In contrast, the marine environment is as rich, or richer, than other comparable habitats elsewhere, with the exception of coral reefs (De Broyer et al., 2014; Peck, 2018).

The marine, freshwater and terrestrial environments south of latitude 60°S lie within the Antarctic Treaty area and are governed through the Antarctic Treaty System (see: https://www.ats.aq/index_e.htm). The Protocol on Environmental Protection to the Antarctic Treaty (agreed 1991) designates Antarctica as a ‘natural reserve, devoted to peace and science’ and, to protect the environmental and scientific values of the continent's biodiversity, prohibits the introduction of nonsterile soil and non‐native species without a permit from a Treaty Party government authority (Hughes & Pertierra, 2016). The issue of inadvertent non‐native species introductions has received academic and policy attention since the Protocol entered into force in 1998 but substantial gaps in research, policy and practice still remain (Chown et al., 2012; Committee for Environmental Protection [CEP], 2017; Frenot et al., 2005; Hughes & Convey, 2010; Hughes & Pertierra, 2016).

### 1.1 Human activity, pathways and vectors

The APR has experienced increasing human activity and an expanding human footprint since it was first visited around 200 years ago (Headland, 2009; Pertierra, Hughes, Vega, et al., 2017). Today, a high propagule pressure (i.e. the number of individuals of a species introduced to a location to which they are not native) results from the rapidly growing numbers of people visiting and volumes of cargo being imported to the APR each year (Key, 2018; Lee & Chown, 2009a). Governmental operators from 18 nations located in Europe, Asia and North and South America have established over 50 research stations and facilities in this region over the last 115 years, and more are planned, thereby increasing the probability of non‐native species arriving from across the globe (Council of Manager of National Antarctic Programs [COMNAP], 2017). Both shipping and rapid air links connect South American and the South Atlantic departure ports to research stations across the APR. While ships may take 2 days or more to cross the Drake Passage to access the APR, aircraft can make the journey in only a few hours, thereby increasing the probability of species surviving the transport process (Hughes, Lee, Ware, Kiefer, & Bergstrom, 2010). Tourism is also increasing rapidly, with almost 42,000 tourists landing at visitor sites during the 2017/18 summer season, predominantly from cruise ships, with these numbers predicted to increase both with recovery from the recent global financial crisis and as the Asian tourist market expands (Bender, Crosbie, & Lynch, 2016; IAATO, 2018). Some sites in the APR of historic or wildlife interest can receive over 20,000 visitors per year (IAATO, 2018; Pertierra, Hughes, Vega, et al., 2017). While the tourism industry has been proactive in the development and implementation of biosecurity practices (IAATO, 2019), an understanding of the level of biosecurity employed by National Antarctic Programmes is less easily obtained (COMNAP, 2008).

Recent and ongoing eradications of introduced plants (Galera et al., 2017, 2019; Molina‐Montenegro et al., 2012; Pertierra, Hughes, Tejedo, et al., 2017) mean that most known remaining macroscopic Antarctic non‐native species are terrestrial invertebrates (Hughes, Pertierra, et al., 2015). However, there are increasing reports of non‐native plants and invertebrates in the APR, almost exclusively in the vicinity of research stations and visitor sites (Hughes, Pertierra, et al., 2015; Molina‐Montenegro et al., 2012; Volonterio, Leon, Convey, & Krzeminska, 2013). There is poor understanding of the rate of non‐native species arrival and establishment across marine, freshwater and terrestrial environments due to a general lack of monitoring (Enríquez et al., 2019; Hughes & Pertierra, 2016; McGeoch, Shaw, Terauds, Lee, & Chown, 2015). Nevertheless, non‐native invertebrates have been found in several locations where soil samples have been taken, suggesting that actual levels of introductions may be greater than currently documented (e.g. see Downie, Convey, McInnes, & Pugh, 2000; Russell et al., 2013). In particular, high human visitation of small geothermal areas on Deception Island can create literal ‘hot spots’ for non‐native species establishment, and the island contains the highest number of known non‐native species in Antarctica (Chown et al., 2012; Enríquez et al., 2019; Greenslade, Potapov, Russell, & Convey, 2012; Hughes, Pertierra, et al., 2015; Longton, 1966; Pertierra, Francisco, Benayas, Smith, & Hughes, 2018; Skottsberg, 1954).

Numerous potential pathways exist for terrestrial non‐native species introductions into the APR (Hughes & Convey, 2010; Key, 2018). Chown et al. (2012) examined the importation of plant propagules into Antarctica in association with the clothing and personal equipment of tourists and other personnel on ships and aircraft. Cargo and associated packing material are also a major vector for propagule importation into Antarctica (Chwedorzewska, Korczak‐Abshire, Olech, Lityńska‐Zając, & Augustyniuk‐Kram, 2013; Houghton et al., 2016; Hughes, Misiak, Ulaganathan, & Newsham, 2018). The delivery of cargo and building materials for the construction of Halley VI Research Station on the Brunt Ice Shelf (located to the east of the APR) resulted in the importation of an estimated 5,000 seeds representing 34 taxa (Lee & Chown, 2009b). Vehicles can also present a risk, with one incident introducing over 132 kg of soil containing viable non‐native vascular plants, bryophytes, insects, microinvertebrates, nematodes, fungi, bacteria, seeds and moss propagules on four construction vehicles (Hughes, Convey, Maslen, & Smith, 2010; Hughes et al., 2018). Fresh food importation can also transport non‐native species, including invertebrates and microbial plant and animal pathogens, on the produce or in associated soil or packaging (Hughes, Cowan, & Wilmotte, 2015; Hughes et al., 2011; Roy et al., 2016). Furthermore, Antarctic hydroponic systems may become infested with non‐native microorganisms and invertebrates, which present a risk to local environments should containment measures fail (Bergstrom et al., 2018; Greenslade et al., 2012; Volonterio et al., 2013). Given the great diversity of pathways and origin of visitors, ships and aircraft accessing the APR, unanticipated species may access the region from many different locations. For example, propagules of the grass *Poa annua* originating from both European and South American sources were introduced independently and established at Arctowski Station on King George Island, South Shetland Islands (Chwedorzewska, 2008). Non‐native marine species may be introduced to Antarctica adhered to vessel hulls, sea chests and intake ports as fouling species, and within ballast water, although propagule pressure via these pathways is not well understood (Lewis, Hewitt, Riddle, & McMinn, 2003; McCarthy, Peck, Hughes, & Aldridge, 2019).

The risk to the biodiversity of the APR is not limited to invasive species originating from outside of the area, but also to the transfer of species native or endemic to one region of Antarctica to another (Hughes et al., 2019; Vyverman et al., 2010). Six distinct biogeographic regions have been identified within the APR, alongside another 10 regions in continental Antarctica, and at least a further 10 comparable distinct areas can be recognized in the various sub‐Antarctic islands (Chown & Convey, 2016; Terauds et al., 2012; Terauds & Lee, 2016). Personnel and cargo movements occurring between these regions may result in the redistribution of endemic or genetically distinct native species, or further dispersal of established non‐native species that originated outside Antarctica (Hughes et al., 2019), as several nations have multiple research stations located in different biogeographic regions within the APR (Figure 1; Lee & Chown, 2011).

### 1.2 Climate change

Unlike much of the rest of Antarctica, the APR experienced substantial warming in the second half of the 20th century, resulting in the retreat of glaciers and complete or partial collapse of many of the region's ice shelves (Cook, Fox, Vaughan, & Ferrigno, 2005; Mulvaney et al., 2012; Turner et al., 2009). Despite a recent pause in the warming trend, global ‘business as usual’ greenhouse gas emission scenarios suggest that the APR will again be subject to rapid warming by 2100 (Turner et al., 2016). This could lead to up to a threefold increase in the area of ice‐free ground in the northern Antarctic Peninsula, resulting in greater connectivity of existing biological communities and potentially creating new habitat both for native biota and new arrivals (Duffy & Lee, 2019; Lee et al., 2017). Distinguishing species introduced by natural and anthropogenic mechanisms will present a substantial challenge (Hughes & Convey, 2012). Many areas of the APR may be vulnerable to invasion by species originating from comparable environments that experience low temperatures, such as the Arctic or high altitude areas (Chown et al., 2012). In a study limited to terrestrial species, Duffy et al. (2017) assessed the climate suitability of the Antarctic and the sub‐Antarctic islands for 69 of the recognized worst globally invasive non‐native species and 24 non‐native insect and plant species that have already established in the region under the RCP8.5 climate scenario. They demonstrated that climate may provide some protection against species establishment in continental Antarctica, but that the APR was vulnerable to invasion by some species that had already established or were invasive on the sub‐Antarctic islands. Looking ahead to 2050 and 2100, the APR remained the most threatened region of Antarctica, with more southerly areas becoming suitable for colonization later in the century.

Within the marine environment, there have been some major recent physical changes, mainly in reductions of marine ice over the continental shelf. In particular, in the past 50 years, seasonal sea ice cover has significantly declined at the west of the Antarctic Peninsula (Parkinson, 2019; Stammerjohn, Massom, Rind, & Martinson, 2012), with coincident considerable marine biodiversity changes (Barnes, 2015) due to marine terminating glacier retreat (Sahade et al., 2015) and ice shelf collapses (Ingels, Aronson, & Smith, 2018; Peck, Barnes, Cook, Fleming, & Clarke, 2010). These changes open up new areas and habitats (e.g. fjords) for colonization, and thus increase the diversity of opportunities for potential invaders.

Different regions of Antarctica may be subject to differing levels of threat from non‐native species due to (a) variation in concentration and extent of human activity, (b) different pathways for introduction, (c) location of source populations, and (d) current and predicted climatic conditions. Stimulated by these considerations, a regional approach was adopted to carry out a horizon scanning of potential invasive species in the APR. Expert opinion and consensus approaches (Roy et al., 2014, 2019) were used to develop a ranked list of potentially invasive non‐native species, currently absent, that are considered likely to arrive, establish and have an impact on native biodiversity and ecosystems over the next decade. The exercise also aimed to identify high‐risk pathways to inform the development of effective biosecurity measures.

## 2 METHODS

Taxonomic and Antarctic experts from nine nations (Argentina, Austria, Belgium, Cyprus, Ireland, Italy, Spain, the United Kingdom [including its Overseas Territories of the British Antarctic Territory, Falkland Islands, South Georgia & the South Sandwich Islands, and Tristan da Cunha] and the United States) met for a workshop in Cambridge, United Kingdom (22–25 October 2018), to undertake a horizon scanning exercise to identify the species that present the highest risk to biodiversity and ecosystems in the APR over the next 10 years. The area under consideration largely coincided with the area recognized as the maritime Antarctic biogeographic zone (Convey, 2017). This exercise was undertaken to fulfil one of the identified requirements of the Antarctic Treaty Consultative Meeting (ATCM; the policy‐making body of the Antarctic Treaty System) as detailed in the Committee for Environmental Protection Non‐native Species Manual (see https://www.ats.aq/documents/ATCM40/att/atcm40_att056_e.pdf).

We used an adapted version of the consensus method (Roy et al., 2014, 2019; Sutherland, Fleishman, Mascia, Pretty, & Rudd, 2011) for a horizon scanning approach, to derive a list of potential invasive non‐native species likely to have high impact in the APR. The approach involved the following steps.

### 2.1 Step 1: Establishment of thematic groups

Experts were placed within four broad thematic groups (marine species, terrestrial and freshwater vascular plants, terrestrial and freshwater invertebrates, terrestrial and freshwater vertebrates). The experts were selected to provide representation across the Antarctic region and ensure sufficient knowledge across taxonomic groups and environments. Each group included four or more people, with size varying, as some experts moved between groups (Table S1). A leader was assigned to each group with the role of coordinating and recording activities and facilitating discussion between group members before, during and after the workshop.

### 2.2 Step 2: Compilation of preliminary lists of potential invasive non‐native species

In advance of the workshop, each thematic group was asked to assemble preliminary lists of potentially invasive non‐native species that they considered the highest risk with respect to the likelihood of arrival, establishment and the magnitude of their potential negative impact on biodiversity and ecosystem services, within the APR over the next 10 years. The lists were compiled from a combination of systematic literature searches (including academic journals, risk assessments, reports, authoritative websites and other ‘grey’ literature), querying of invasive non‐native species databases (e.g. CABI ISC, GISD, EPPO, GRIIS, WoRMS and WRiMS), and their own expert knowledge. The geographic scope of the search for potentially invasive non‐native species was worldwide. Only species currently considered absent, including those that may have been present, but which are reported to have been extirpated, in the region were included. The temporal scope of the horizon scanning exercise was that only species likely to arrive in the next 10 years should be included, thus limiting to some degree the relevance of longer term climate change projections.

The scope of the exercise was further refined based on a number of exclusions:

1. Species that arrive from their native range by natural spread/dispersal without human intervention, possibly in response to changing ecological conditions or climate change; however, ‘unaided’ dispersal pathways from other invasive ranges within Antarctica were also considered.
2. Parasites that cause animal diseases (including to wildlife).
3. Microorganisms and macroscopic fungi.

The consultation among experts within thematic groups was completed both through e‐mail in advance of the workshop (over 4 weeks) and through smaller thematic group discussions during the workshop. Leaders of each of the thematic groups collated the lists of invasive non‐native species, received from the experts within their group, into a single provisional list.

### 2.3 Step 3: Scoring of species

Experts worked together to agree scores for each species within their thematic group for their separate likelihoods of: (a) arrival, (b) establishment, and (c) magnitude of the potential negative impact on biodiversity or ecosystems. A five‐point scale from 1 = very low to 5 = very high was adopted to achieve an appropriate balance between accuracy and resolution (see Roy et al., 2019, for further details). The scores from each expert, within each thematic group, were then compiled and discussions within the thematic groups (at the workshop) led to an overall agreed impact and confidence score for each species with respect to these aspects of their potential invasions. Confidence levels (low, medium or high) were attributed to each score to help focus discussions and refine the list of species, but were not used formally within the consensus building across all thematic groups.

While acknowledging that the scores were for the purposes of ranking only, and not to be interpreted metrically, an overall risk score for each species was calculated as the product of the individual scores for arrival, establishment and impact. With a three‐criterion, five‐point scoring system, this produces a maximum score of 125.

### 2.4 Step 4: Expert (consensus) approach

The aims of the 4 day workshop were to complete the scoring of species within thematic groups and collaboratively rank the species to achieve a priority list, based on the magnitude of potential impact to the APR environment. On the final day of the workshop, all the species lists from across the thematic groups were collated into a single overall list. At this stage, there were 103 non‐native species listed. Experts were invited to justify their scores in comparison to those of other groups and to increase the alignment of results among groups through a further round of review and moderation of the lists. Changes to overall rankings for individual species were made only after hearing the evidence from appropriate experts, full discussion and, if needed, majority voting. The end‐result was an agreed ranked list of potential invasive non‐native species, derived through discussion and broad consensus; these species were considered to represent a high probability of arrival, establishment and magnitude of impact on biodiversity and ecosystem function for the APR.

## 3 RESULTS

A total of 103 species were identified as relevant for further review through the consensus workshop (Table S2). From this list, 13 non‐native species were identified as presenting a high risk (i.e. with a score ≥50) of invading the APR and adversely affecting biodiversity or ecosystems in the next 10 years (Table 1). All workshop participants agreed that the list represented the outcome of the consensus approach.

**Table 1:** Results of the horizon‐scan exercise to identify invasive non‐native species likely to threaten biodiversity and ecosystems in the Antarctic Peninsula region. Species allocated the highest score (A*B*C) are considered most likely to become invasive within the region

No.	Species	Common name	Taxonomy	Broad group	Functional group	Native range	Pathways of arrival	Comment on impact	Arrival (A)	Establishment (B)	Biodiversity impact (C)	A*B*C	Confidence
1	*Mytilus chilensis*	Chilean mussel	Mollusca: Bivalvia	Marine invertebrate	Filter‐feeder	NE, NW Atlantic, Mediterranean	Hull	No Mytilidae in region, so major impact on native species; filter‐feeder alters community composition	5	5	5	125	M
2	*Mytilus edulis* a	Common blue mussel	Mollusca: Bivalvia	Marine invertebrate	Filter‐feeder	NE, NW Atlantic	Hull	No Mytilidae in region, so major impact on native species; filter‐feeder alters community composition	5	5	5	125	M
3	*Protaphorura fimata*	Springtail	Collembola: Poduromorpha: Onychiuridae	Terrestrial invertebrate	Detritivore	Palaeartic; introduced to sub‐Antarctic	Food, luggage, container, machinery	Potential to alter community structure through competition	4	5	5	100	H
4	*Nanorchestes antarcticus*	Mite^b^	Acari: Prostigmata	Terrestrial invertebrate	Predator	Continental Antarctica; not APR	Container, machinery	Increase ecosystem complexity Environmental change: possible consequences for the life histories of Antarctic terrestrial biota in APR	4	5	5	100	H
5	*Halicarcinus planatus*	Decapod	Arthropoda: Hymenosomatidae	Marine invertebrate	Omnivore/detritivore	Sub‐Antarctic, including Pacific Ocean up to southern Peru	Ballast, hull	Outcompete native species and alter community composition	5	5	4	100	L
6	*Ciona intestinalis*	Sea vase	Chordata: Ascidiacea	Marine invertebrate	Filter‐feeder	Europe	Hull	Reduce local species diversity and alter community assembly processes to fundamentally change sessile community composition	5	5	4	100	L
7	*Leptinella scariosa*	A Buttonweed	Asterales: Asteraceae	Terrestrial plant	Primary producer	Southern Chile, southern Argentina, Falkland Islands	Clothing, luggage, machinery, vehicle, container	Increase ecosystem complexity; potential to alter community structure through competition	4	5	4	80	M
8	*Botryllus schlosseri*	Colonial Ascidian	Chordata: Ascidiacea	Marine invertebrate	Filter‐feeder	West Pacific	Hull	Overgrows shellfish and other sessile invertebrate species	4	4	4	64	L
9	*Carcinus maenas*	European Shore Crab	Arthropoda: Malacostraca	Marine invertebrate	Omnivore	Atlantic Europe, the western Baltic and west Africa to Mauritania	Hull, ballast	Outcompetes native species and can alter community composition	4	4	4	64	L
10	*Undaria pinnatifida*	Asian kelp	Phaeophyta: Laminariales	Marine algae	Primary producer	Asia and Russia	Hull	Potential to reduce native species diversity through competition	4	3	5	60	L
11	*Leptinella plumosa*	A Buttonweed	Asterales: Asteraceae	Terrestrial plant	Primary producer	Antipodes, Campbell, Auckland, Heard, Macquarie, Kerguelen, Crozet and Marion Islands	Luggage, machinery, vehicle, container	Increase ecosystem complexity; potential to alter community structure through competition	3	5	4	60	M
12	*Chaetopterus variopedatus*	Parchment worm	Annelida: Chaetopteridae	Marine invertebrate	Filter‐feeder	Unknown	Hull, ballast	Potential to outcompete native species and alter community assembly	3	5	4	60	L
13	*Mytilus galloprovincialis*	Mediterranean mussel	Mollusca: Bivalvia	Marine invertebrate	Filter‐feeder	Mediterranean	Hull	No Mytilidae in region, so major impact on native species; filter‐feeder alters community composition	5	2	5	50	L

a Note the taxonomy of this *Mytilus* is unresolved and represents a worldwide *Mytilus edulis* complex of mussels.

b Coming from other Antarctic regions.

### 3.1 Taxonomic and environmental breadth

The 13 non‐native species identified as presenting the highest risk included eight marine invertebrates (with four marine molluscs included), one marine alga, two terrestrial invertebrates and two vascular plants (Table 1). No vertebrates were considered to represent a risk over the time span of 10 years.

### 3.2 Native range and pathways

The native ranges of the 13 species span regions from the Northern and Southern Hemispheres. The most likely pathway for introduction of the majority of marine species is anticipated to be on the hulls of ships and, thus, the distances over which the species could be transported will be linked to global shipping routes. The risk of introductions of marine species associated with ship ballast water was considered low due to existing Antarctic Treaty System and IMO regulations which stipulate ballast water exchange at the Polar Front (International Maritime Organisation Marine Environment Protection Committee, 2007). The threat of introductions is likely to diminish further once all relevant vessels visiting Antarctica comply with more stringent ballast water management regulations that entered into force in 2017 (International Maritime Organisation [IMO], 2004). The most likely pathways for the introduction of terrestrial invertebrates and plants include transported clothing and luggage of visitors, cargo, fresh produce and vehicles which can carry propagules or whole organisms, often within inadvertently imported mud and soil.

## 4 DISCUSSION

Some of the least impacted ecosystems globally are located within Antarctica, including those least invaded by non‐native species (Hughes, Pertierra, et al., 2015); however, the APR is under substantial and imminent risk of invasion (Chown et al., 2012). Here we discuss the findings of the horizon scanning exercise according to the four thematic groups examined.

### 4.1 Marine species

We suggest that, while terrestrial environments may be at some risk from plants and invertebrates, the greatest immediate threat to the APR is likely to come from invasive marine species, with 9 of the 13 species identified as high risk being marine. Three of the high‐risk species were *Mytilus* bivalve molluscs (mussels, see Table 1), which are well documented as non‐native invaders globally (see World Register of Introduced Marine Species [WRiMS] database [see http://www.marinespecies.org/introduced/]). *Mytilus* species grow quickly, can smother shores and shallow subtidal regions reducing diversity and impacting on ecosystems, and have been recorded travelling through the Southern Ocean on ship hulls (Lee & Chown, 2007; Lewis et al., 2003). There has been an example of a single individual of a non‐native *Mytilus* species recorded from the sub‐Antarctic island of South Georgia (Ralph, Maxwell, Everson, & Hall, 1976), which lies only 800 km north of the APR. It is becoming increasingly apparent, however, that *Mytilus* species cannot be distinguished reliably from each other without the use of molecular tools, which could present a problem for monitoring and surveillance (although at the coarsest level, observation of any *Mytilus* present would signal arrival and establishment of a new non‐native species). Other non‐native species recognized as presenting the highest risk include common coastal crabs (e.g. *Halicarcinus planatus*), albeit that this group is known to have low temperature physiological limitations. True crabs (which, like mussels, have no native equivalents in the Southern Ocean), ascidians (e.g. *Ciona intestinalis*), macroalgae, polychaete worms and bryozoans are well known as invasive elsewhere and can be abundant at gateway ports to the APR, such as Ushuaia (Argentina), Punta Arenas (Chile) and Stanley (Falkland Islands; Lewis et al., 2003). Nevertheless, the lack of information available on the likelihood of establishment and potential impact of the identified marine species on native Antarctic biodiversity means that the confidence levels assigned to these assessments were rather low (Table 1). If predicted warming occurs in the shallows around the APR, native marine species may change in geographic and bathymetric range and/or become less competitive within parts of these ranges, facilitating the establishment and spread of any non‐native species.

It is expected that some colonization will be a consequence of natural processes, such as rafting on kelp or through pelagic life‐stages, which have been caught in eddies of the Antarctic Circumpolar Current (Clarke, Barnes, & Hodgson, 2005; Fraser et al., 2018). However, there is an increasing (and accelerating) diversity and quantity of anthropogenic substrata to foul, such as plastics (Barnes, 2002). Plastic debris in the South Atlantic has increased 100‐fold in the last decade alone (Barnes et al., 2018), much of it is colonized by biota. However, there is virtually no assessment of monitoring of this new potential transfer route or recognition of it as a threat, possibly because any solution does not lie within the region and rather requires pressure to reduce and recycle at source. Our horizon scanning focused on anthropogenic pathways, and for marine species the major pathway is anticipated to be ships, although the plastic debris pathway should not be underestimated.

The ATCM and IMO have agreed ballast water exchange protocols for ships entering Antarctic waters (ATCM XXIX Resolution 3 [2006]; International Maritime Organisation Marine Environment Protection Committee, 2007). However, many ships accessing the region do not yet comply with the more stringent ballast water regulations that entered into force in 2017, requiring ships to treat or exchange ballast water, and so this pathway also continues to represent some degree of threat (IMO, 2004). Non‐native species may persist on cargo tenders, within sea chests, bow thrusters or water intake pipes on tourist, fishing, military and national operator vessels active in the region (Lee & Chown, 2009b, 2009c; Lewis et al., 2003; Lewis, Riddle, & Hewitt, 2004). Southward transport of fouling species on vessel hulls presents a risk, as overwintering or operation of vessels in more northerly warm water regions creates the potential for the development of substantial fouling communities, including globally invasive marine species (Lewis et al., 2003). Added to this, a decline in sea ice to the west of the Antarctic Peninsula (Parkinson, 2019) means that non‐native hull biofouling species are less likely to be scoured from vessels by ice abrasion, albeit many vessels actively avoid entering areas of sea ice (Hughes & Ashton, 2017; Ware et al., 2014). Propagule pressure may be highly variable due to differences in the level of invasion of vessel home ports, ship antifouling practices and the routes and duration of voyages to the Antarctic (Lewis et al., 2003; McCarthy et al., 2019). However, the lack of regular and routine defouling of ships (possibly due to the perceived expense and impact on itineraries) means this pathway may present one of the greatest threats to biodiversity in the nearshore environment around the APR and beyond.

Information regarding the movement of national operator and military vessels is not readily available; however, data for the highest concentrations of tourist ship traffic have been reported from the Lemaire and Neumayer Channels, with increasing activity in the Gerlache Strait (Bender et al., 2016). Several popular landing sites on the northern Peninsula and South Shetland Islands (e.g. Cuverville Island and Half Moon Island, Goudier Island, Neko Harbour and Whalers Bay) typically experience more than 100 ship visits during the summer season (IAATO, 2018). Cruise vessels generally anchor or hold position for several hours while tourist landings occur, generating the opportunity for non‐native marine species to be introduced to the local nearshore environment. Furthermore, while tourist visits to Peninsula sites may be frequent but rapid, military or national operator vessels may be present at locations (which are concentrated in the South Shetland Islands and northern Peninsula; see Figure 1) for prolonged periods to facilitate station resupply or support research activities, thereby creating opportunities for the dispersal of non‐native fouling species.

### 4.2 Terrestrial and freshwater invertebrates

While marine species dominate the list, key terrestrial and freshwater invertebrates were identified that could pose a threat by altering ecosystem function within this unique environment. Indeed, invertebrates have already been introduced and established on the South Orkney Islands (Signy Island) from South Georgia (Bartlett, Convey, Pertierra, & Hayward, 2019; Hughes & Worland, 2010). More specifically, the non‐native flightless chironomid *Eretmoptera murphyi* may have increased soil nutrient cycling rates on Signy Island by up to nine times compared to the indigenous fauna (Hughes, Worland, Thorne, & Convey, 2013). This species is predicted to have a suitably flexible physiology to enable it to both expand its distribution further on Signy Island and to colonize sites on the western Antarctic Peninsula up to 750 km further south (Hughes et al., 2013; Worland, 2010). Within the APR, the majority of existing introductions have been microarthropods, such as Collembola and Acari, with no eradication attempts made or likely to be practicable (Hughes, Pertierra, et al., 2015). Furthermore, few if any data exist on transfer or establishment of other microinvertebrate groups that are major elements of terrestrial communities across the region (i.e. Nematoda, Tardigrada, Rotifera). It should be emphasized that the terrestrial invertebrate species mentioned in Table 1 are ‘representative’—at present explicit evidence does not exist to enable differentiation across a wide diversity of representatives of these groups occurring naturally or already introduced in the wider sub‐Antarctic (especially South Georgia). However, Greenslade et al. (2012), considering recent establishment of non‐native Collembola on Deception Island, noted that several of the species now present are identified as high risk in the assessment that Greenslade and Convey (2012) applied to the sub‐Antarctic islands, again, highlighting the heightened risk associated with parts of the APR. Similarly, Russell et al. (2013) have reported several non‐native mite and springtail species recently established in the South Shetland Islands. Invertebrates may also be readily transported between distinct biogeographic regions that exist within the Antarctic and sub‐Antarctic islands (Chown & Convey, 2016; Frenot et al., 2005; Terauds et al., 2012; Terauds & Lee, 2016). In the horizon scan this is represented by the inclusion of the continental Antarctic endemic *Nanorchestes antarcticus* as a potential threat to the APR through human‐mediated transfer and colonization. In this example, ice‐free locations within the APR with climatic conditions similar to those found within the species native range may be particularly vulnerable (Hughes et al., 2019; Table 1).

A particular concern relates to the possibility of introduction of ‘ecosystem engineer’ species that bring new ecosystem functions, or drastically alter their magnitude. For instance, detritivore species such as *Protaphorura fimata* (Table 1) or other Collembola species might cause a great impact in the nutrient cycling of terrestrial ecosystems. The introduction of *Eretmoptera murphyi* already provides an example of this, being a detritivore capable of a greater magnitude of peat recycling than the entire native invertebrate community where it occurs (Hughes et al., 2013), while the same is also likely to be the case with the boreal trichocerid *Trichocera maculipennis* on the South Shetland Islands (Potocka & Krzemińska, 2018; Volonterio et al., 2013). At present, no climate matching modelling studies have been attempted, but there must be concern that major new predatory guilds such as carabid beetles (introduced to and expanding rapidly on sub‐Antarctic South Georgia and Îles Kerguelen) and earwigs may be capable of making the jump to more benign areas of the APR, such as parts of the South Orkney and South Shetland Islands, or indeed to the fragile geothermal habitats represented in the South Shetlands by Deception Island.

### 4.3 Terrestrial and freshwater vascular plants

Historically, the majority of vascular plants introduced to the Antarctic Treaty area have been recorded within the APR (Frenot et al., 2005; Hughes, Pertierra, et al., 2015; Smith, 1996). Deliberate experimental introductions of non‐native vascular plants were undertaken during the 1950s and 1960s, but the transplanted species were either unable to establish or were later removed (Corte, 1961; Edwards, 1980; Smith, 1996). Plants that have established, however, have mostly been weedy cosmopolitan species, with species from the genus *Poa* dominating (Cuba‐Diaz, Troncoso, Cordero, Finot, & Rondanelli‐Reyes, 2012; Galera et al., 2017; Molina‐Montenegro, Bergstrom, Chwedorzewska, Convey, & Chown, 2019; Molina‐Montenegro et al., 2012; Peter, Buesser, Mustafa, & Pfeiffer, 2008; Smith & Richardson, 2010). Recent efforts have removed *P. annua* from several affected APR locations (Malfasi, Convey, Zaccara, & Cannone, 2019; Molina‐Montenegro et al., 2012, 2015); however, the presence of high numbers of *P. annua* plants and its persistent seed banks around Admiralty Bay, King George Island, remain a management issue despite on‐going eradication efforts (Galera et al., 2017; 2019; Wódkiewicz, Ziemiański, Kwiecień, Chwedorzewska, & Galera, 2014). In 2015, *Poa pratensis* was successfully eradicated from the APR, but the grass scored highly on our horizon scanning list as previously present species are highly likely to reoccur, either from seedbanks or through rearrival as a contaminant of people, vehicles and cargo, and their establishment and impact potential has already been proven (Molina‐Montenegro et al., 2012, 2015; Pertierra, Hughes, Tejedo, et al., 2017).

Other vascular species identified through our scan included two *Leptinella* species (Asteraceae). *Leptinella* is a genus of low‐growing, hemicryptophytic plants with plumed seeds. Transport along relevant pathways for the Antarctic (e.g. as contaminants of humans and their luggage, machinery, vehicles, containers) seems possible, and several members of the genus are abundant throughout higher latitudes in the Southern Hemisphere, including on sub‐Antarctic islands from where scientific personnel are regularly exchanged with the APR (Floyd, 2018; Lloyd, 1972; Turner, Scott, & Rozefelds, 2005). They are also found in areas, such as Punta Arenas, Ushuaia and the Falkland Islands, from where much ship traffic departs for the Antarctic Peninsula (Lloyd, 1972; Upson & Lewis, 2014).

A majority of the other vascular plants on our list were grasses, particularly perennial grasses with rhizomatous or stoloniferous growth, potentially preadapting them for a climatically ameliorating APR (Callaghan, 1988; Callaghan & Emanuelsson, 1985). For example, *Schedonorus arundinaceus*, *Agrostis stolonifera*, *Anthoxanthum odoratum*, *Deschampsia cespitosa*, *Nardus stricta*, *Elymus repens* and *Arrhenatherum elatius* were all selected as being more likely than most other vascular plant species to arrive and establish in warmer parts of the APR. Arrival of these species along pathways from other locations was considered possible due to their wide distribution, and to the regular movement of scientists and equipment between source populations and the APR; in addition, several of these species are already established in parts of the sub‐Antarctic region or nearby (see Duffy et al., 2017; Frenot et al., 2005). In a similar vein, the widely distributed global weed *Plantago major* also has successfully established in the High Arctic, and is regularly found attached to visitor's clothing and cargo (e.g. Chown et al., 2012; Kalwij, Robertson, & van Rensburg, 2015). However, these species were not included in the list of highest risk species (Table 1) as the potential for them to have significant impacts on Antarctic biodiversity within 10 years was still considered to be low, largely due to an expectation that their spread potential within that time period would be minimal.

Although it is challenging to make predictions of this nature, we have attempted to ensure that the selected vascular plant species cover a range of types thought relevant for the protection of Antarctic biodiversity by focusing on known pathways, local abundances in source countries, environmental tolerances, species' propagule types and evidence from other sub‐Antarctic and Arctic regions. Given the low native plant diversity within the Antarctic, the pool of potential arrivals is clearly very large, even when just considering nearby areas with similar or near‐similar climates (such as the sub‐Antarctic islands). Choosing those native species from sub‐Antarctic islands, South Atlantic islands or South America, all of which are well‐connected to the APR, is a particular challenge, particularly given that Patagonian species have arrived through unknown pathways within the recent past (Smith & Richardson, 2010). Finally, we have not considered potential threats from non‐native bryophyte or lichen species within the current exercise. Extending the current approach to these taxa would be useful, although the fact that many of the species described are difficult to determine in the field (Ochyra, Smith, & Bednarek‐Ochyra, 2008) may make this a challenging task, further complicated as on‐going survey work is highly likely to extend the known distributions of many taxa across the region. For example, one of the most invasive bryophytes of the Northern Hemisphere, *Campylopus introflexus* (native to many parts of the Southern Hemisphere) has been reported as native to some sub‐Antarctic islands, but not to the Antarctic itself. However, this species was previously considered to be native to the Antarctic on Deception Island, until recent reviews of voucher material demonstrated that the specimen had been misidentified (Ochyra et al., 2008).

### 4.4 Vertebrates

Introduced vertebrates (including, for example, rats, mice, rabbits and cats) have caused dramatic adverse effects upon communities globally, including several sub‐Antarctic islands (Frenot et al., 2005). However, it was considered unlikely that any introduced vertebrates would establish in the APR in the next 10 years, due to a combination of the cold climatic conditions and lack of suitable habitat and year‐round food sources, and the complete ban on their intentional import to the region under the rules of the Antarctic Treaty System. While various vertebrates species were introduced to several sub‐Antarctic islands, including as a consequence of sealing and whaling in the 19th and 20th centuries, equivalent activities undertaken within the APR did not result in vertebrate establishment, supporting the suggestion that the environmental conditions are probably unsuitable for these species (Frenot et al., 2005). There have been instances of the presence of dogs and birds on yachts and other vessels visiting the region, although there are few known cases of these landing (France, 2018; Headland, 2012). There has also been one report of an inadvertent release of a rat onto King George Island, which was subsequently found dead (Peter et al., 2008). Nonetheless, vertebrates could again arrive in future as stowaways or as a result of intentional introductions and stringent controls and biosecurity measures are required to reduce the risk. The low establishment potential for bird species in the APR, due predominantly to harsh climatic conditions, is demonstrated by records of the rapid demise of a range of vagrant bird species that arrived in the area (Petersen, Rossi, & Petry, 2015).

### 4.5 Recommendations

The APR, more than any other region in the Antarctic Treaty area, is at risk from invasive non‐native species. In particular, this horizon scanning exercise has highlighted microinvertebrates and marine species as of particular concern to Antarctica. Perhaps unsurprisingly, the invasive non‐native species identified as a potential threat to the APR are a striking contrast to those derived through similar horizon scanning exercises focusing on the Northern Hemisphere (principally Europe; Carboneras et al., 2018; Peyton et al., 2019; Roy et al., 2014, 2019).

Within the Antarctic Treaty area, there has been some progress in the development of internationally endorsed biosecurity procedures, with the production of a Non‐native Species Manual by the CEP (2017) and biosecurity checklists by the COMNAP (2010). Nevertheless, the level of biosecurity implementation and effectiveness of surveillance practices by national operators, where employed, are variable and not known in detail. Mechanisms and practices to reduce the risk of marine non‐native species introductions on ship hulls are likely to be minimal if present at all. In terms of environmental management, the employment of consistently high biosecurity standards by national and tourism operators when transporting personnel and cargo into the APR either by air or sea, and between the distinct biogeographic regions therein and beyond, comes at a cost. However, this is likely to be trivial compared to the substantial expense of operating research stations, ships and aircraft in the region, or eradicating an introduced species once established (Hughes & Pertierra, 2016; Hughes, Pertierra, et al., 2015). We, therefore, recommend the further development and consistent application of effective biosecurity measures across all national operators and the tourism and fishing industries, and for surveillance and appropriate rapid response action for introduced species, such as those identified during this horizon scanning exercise. Furthermore, the conservation objectives of the ATCM would be usefully served by the adoption of this methodology across the other regions of Antarctica.

## CONFLICT OF INTEREST

The authors have no conflict of interest to declare.

## AUTHOR CONTRIBUTIONS

HER conceived the approach and led the horizon scanning exercise. KAH led the writing of the manuscript with HER, OLP, JP, PC and DKAB providing substantial contributions. All authors contributed to the prioritization exercise including compilation of lists and rapid impact assessments and commented on the writing of the manuscript.
