# Supplementary material for: Invasive non‐native species likely to threaten biodiversity and ecosystems in the Antarctic Peninsula region
Source: Glob Chang Biol. 2020 Jan 13;26(4):2702–16. doi: 10.1111/gcb.14938 (PMC7154743; doi:10.1111/gcb.14938)
Supplement: Supplementary file 1 [file GCB-26-2702-s001.docx]

**Supplementary Information**

Table S1. List of experts participating in the horizon scanning exercise, including expertise contributed

| **Name** | **Organisation** | **Expertise** |
| --- | --- | --- |
| Helen Roy | Centre for Ecology & Hydrology | Project lead, invertebrates |
| Jodey Peyton | Centre for Ecology & Hydrology | Project manager, botany |
| Katharine Turvey | Centre for Ecology & Hydrology | Data management |
| Oliver Pescott | Centre for Ecology & Hydrology | Thematic group lead: Botany |
| Wayne Dawson | Durham University | Botany |
| Pablo González-Moreno | CABI | Botany |
| Jonathan Shanklin | British Antarctic Survey | Botany |
| Tim Adriaens | Research Institute for Nature and Forest | Thematic group lead: Vertebrates |
| Ian Winfield | Centre for Ecology & Hydrology | Vertebrates |
| Stephanie Martin | Government of Tristan da Cunha | Vertebrates |
| Andrea Monaco | Lazio Regional Authority, Rome, Italy | Vertebrates |
| Elena Tricarico | University of Florence | Thematic group lead: Invertebrates |
| Wolfgang Rabitsch | Environment Agency Austria | Thematic group lead: Invertebrates |
| Christopher Malumphy | Fera Science Ltd. | Invertebrates |
| Peter Convey | British Antarctic Survey | Invertebrates |
| Naomi Baxter | Falkland Islands Government | Invertebrates, environmental management |
| Angeliki Martinou | The Cyprus Institute | Invertebrates (human health) |
| Victoria Werenkraut | CONICET | Invertebrates |
| Alexander Vaux | Public Health England | Invertebrates (human health) |
| Elizabeth Cottier-Cook | Scottish Association for Marine Science | Thematic group lead: Marine species |
| David Barnes | British Antarctic Survey | Marine species |
| Mark Belchier | British Antarctic Survey | Marine species |
| Dan Minchin | Marine Organism Investigations | Marine species |
| Simon Morley | British Antarctic Survey | Marine species |
| Kevin Hughes | British Antarctic Survey | Policy, environmental management |
| Katherine Ross | Falklands Conservation | Invertebrates, vertebrates, botany |
| Ross James | Government of South Georgia & the South Sandwich Islands | Invertebrates, vertebrates |
| David Vaughan | British Antarctic Survey | Polar expertise |
| Denise Blake | Falkland Islands Government | Environmental management |
| Kevin Smith | IUCN | Invasion biology |
| Niall Moore | GB Non-Native Species Secretariat | Policy, vertebrates |
| Gillian Key | GB Non-Native Species Secretariat | Pathways |

|  |  |  |
| --- | --- | --- |
|  |  |  |
|  |  |  |
|  |  |  |
|  |  |  |
|  |  |  |
|  |  |  |
|  |  |  |
|  |  |  |
|  |  |  |
|  |  |  |
|  |  |  |
|  |  |  |
|  |  |  |
|  |  |  |
|  |  |  |
|  |  |  |
|  |  |  |
|  |  |  |
|  |  |  |
|  |  |  |
|  |  |  |
|  |  |  |
|  |  |  |
|  |  |  |
|  |  |  |
|  |  |  |
|  |  |  |
|  |  |  |
|  |  |  |
|  |  |  |
|  |  |  |
|  |  |  |
|  |  |  |
|  |  |  |
|  |  |  |
|  |  |  |
|  |  |  |
|  |  |  |
|  |  |  |
|  |  |  |
|  |  |  |
|  |  |  |
|  |  |  |
|  |  |  |
|  |  |  |
|  |  |  |
|  |  |  |
|  |  |  |
|  |  |  |
|  |  |  |
|  |  |  |
|  |  |  |
|  |  |  |
|  |  |  |
|  |  |  |
|  |  |  |
|  |  |  |
|  |  |  |
|  |  |  |
|  |  |  |
|  |  |  |
|  |  |  |
|  |  |  |
|  |  |  |
|  |  |  |
|  |  |  |
|  |  |  |
|  |  |  |
|  |  |  |
|  |  |  |
|  |  |  |
|  |  |  |
|  |  |  |
